# Supplementary material for: Fibroblast growth factor receptor 2 (FGFR2) genetic polymorphisms contribute to fused roots in human molars
Source: PLoS One. 2025 Apr 10;20(4):e0316904. doi: 10.1371/journal.pone.0316904 (PMC11984737; doi:10.1371/journal.pone.0316904)
Supplement: S1 Table — Note: Bold indicates a statistically significant difference (p < 0.05). (DOCX) [file pone.0316904.s001.docx]

| **Haplotype** | | **Frequency (%)** | | **p-value** |
| --- | --- | --- | --- | --- |
|  |  | **Case** | **Control** |  |
| **Maxillary + mandibular fused root (45 combinations)** | | | | |
| rs2162540\|rs2981578\|rs10736303\|rs1078806\|rs11200014\|rs1219648\|rs4752566 | TTGAGAG | 0.07 | 0.01 | **0.016** |
| rs2162540\|rs2981578\|rs10736303\|rs1078806\|rs1219648\|rs4752566 | CCGGAT | 0.03 | 0.006 | **0.043** |
| rs2162540\|rs2981578\|rs10736303\|rs1078806\|rs1219648\|rs4752566 | TTGAAG | 0.04 | 0.01 | **0.049** |
| rs2162540\|rs2981578\|rs10736303\|rs11200014\|rs1219648\|rs4752566 | TTGGAG | 0.07 | 0.02 | **0.037** |
| rs2162540\|rs2981578\|rs1078806\|rs11200014\|rs1219648\|rs4752566 | CCGAAT | 0.03 | 0.001 | **0.007** |
| rs2162540\|rs10736303\|rs1078806\|rs11200014\|rs1219648\|rs4752566 | CGGAAT | 0.03 | 0.002 | **0.011** |
| rs2162540\|rs10736303\|rs1078806\|rs11200014\|rs1219648\|rs4752566 | TGAGAG | 0.06 | 0.01 | **0.013** |
| rs2981578\|rs10736303\|rs1078806\|rs11200014\|rs1219648\|rs4752566 | TGAGAG | 0.05 | 0.01 | **0.019** |
| rs2162540\|rs2981578\|rs10736303\|rs1078806\|rs1219648 | CCGGA | 0.04 | 0.004 | **0.006** |
| rs2162540\|rs2981578\|rs10736303\|rs1219648\|rs4752566 | TTGAG | 0.07 | 0.02 | **0.045** |
| rs2162540\|rs2981578\|rs10736303\|rs11200014\|rs4752566 | TTGGG | 0.07 | 0.02 | **0.026** |
| rs2981578\|rs10736303\|rs1078806\|rs11200014\|rs1219648 | TGAGA | 0.09 | 0.03 | **0.046** |
| rs2981578\|rs10736303\|rs1078806\|rs1219648\|rs4752566 | TGAAG | 0.05 | 0.01 | **0.031** |
| rs2981578\|rs10736303\|rs1078806\|rs11200014\|rs4752566 | TGAGG | 0.05 | 0.01 | **0.029** |
| rs2162540\|rs2981578\|rs10736303\|rs4752566 | TTGG | 0.08 | 0.02 | **0.028** |
| rs2162540\|rs10736303\|rs1078806\|rs11200014 | TGAG | 0.12 | 0.05 | **0.038** |
| rs2162540\|rs10736303\|rs1078806\|rs11200014 | CAAG | 0.04 | 0.12 | **0.032** |
| rs2162540\|rs10736303\|rs1078806\|rs1219648 | CGGA | 0.04 | 0.003 | **0.005** |
| rs2162540\|rs10736303\|rs1078806\|rs1219648 | TGAA | 0.13 | 0.06 | **0.047** |
| rs2162540\|rs10736303\|rs1078806\|rs1219648 | CAAA | 0.04 | 0.13 | **0.023** |
| rs2162540\|rs10736303\|rs1078806\|rs4752566 | CAAT | 0.008 | 0.07 | **0.025** |
| rs2981578\|rs10736303\|rs1078806\|rs4752566 | TGAG | 0.06 | 0.01 | **0.038** |
| rs2981578\|rs10736303\|rs11200014\|rs1219648 | TGGA | 0.09 | 0.04 | **0.048** |
| rs2981578\|rs10736303\|rs1219648\|rs4752566 | TGAG | 0.07 | 0.01 | **0.026** |
| rs10736303\|rs1078806\|rs11200014\|rs1219648 | GAGA | 0.12 | 0.04 | **0.016** |
| rs10736303\|rs1078806\|rs1219648\|rs4752566 | GAAG | 0.06 | 0.02 | **0.043** |
| rs10736303\|rs1078806\|rs11200014\|rs4752566 | GAGG | 0.06 | 0.01 | **0.022** |
| rs2162540\|rs2981578\|rs10736303 | TTG | 0.11 | 0.04 | **0.046** |
| rs2162540\|rs10736303\|rs1078806 | CAA | 0.05 | 0.15 | **0.018** |
| rs2162540\|rs10736303\|rs11200014 | CAG | 0.04 | 0.12 | **0.029** |
| rs2162540\|rs10736303\|rs1219648 | TGA | 0.15 | 0.07 | **0.034** |
| rs2162540\|rs1078806\|rs1219648 | CGA | 0.04 | 0.002 | **0.006** |
| rs2162540\|rs1078806\|rs1219648 | CAA | 0.04 | 0.12 | **0.042** |
| rs2162540\|rs1078806\|rs4752566 | CAT | 0.009 | 0.06 | **0.043** |
| rs2981578\|rs10736303\|rs11200014 | TGG | 0.10 | 0.04 | **0.046** |
| rs2981578\|rs10736303\|rs4752566 | TGG | 0.07 | 0.02 | **0.023** |
| rs2162540\|rs10736303 | TG | 0.24 | 0.15 | **0.047** |
| rs2162540\|rs10736303 | CA | 0.07 | 0.18 | **0.026** |
| rs2162540\|rs1078806 | CA | 0.06 | 0.16 | **0.025** |
| rs2162540\|rs11200014 | CG | 0.06 | 0.15 | **0.040** |
| rs2981578\|rs10736303 | TG | 0.10 | 0.04 | **0.047** |
| rs10736303\|rs1078806 | GA | 0.13 | 0.06 | **0.046** |
| rs10736303\|rs1219648 | GA | 0.18 | 0.08 | **0.012** |
| rs2981578\|rs10736303 | TG | 0.10 | 0.04 | **0.047** |
| rs10736303\|rs1219648 | GA | 0.18 | 0.08 | **0.012** |
| **Maxillary fused root (15 combinations)** | | | | |
| rs2162540\|rs2981578\|rs10736303\|rs1078806\|rs11200014\|rs1219648\|rs4752566 | TTGAGAG | 0.06 | 0.01 | **0.043** |
| rs2162540\|rs2981578\|rs10736303\|rs1078806\|rs1219648\|rs4752566 | TTGAAG | 0.05 | 0.01 | **0.041** |
| rs2162540\|rs2981578\|rs1078806\|rs11200014\|rs1219648\|rs4752566 | CCGAAT | 0.02 | 0.001 | **0.030** |
| rs2162540\|rs10736303\|rs1078806\|rs11200014\|rs1219648\|rs4752566 | CGGAAT | 0.02 | 0.002 | **0.045** |
| rs2162540\|rs10736303\|rs1078806\|rs11200014\|rs1219648\|rs4752566 | TGAGAG | 0.05 | 0.01 | **0.033** |
| rs2981578\|rs10736303\|rs1078806\|rs11200014\|rs1219648\|rs4752566 | TGAGAG | 0.04 | 0.01 | **0.047** |
| rs2162540\|rs10736303\|rs1078806\|rs11200014 | CAAG | 0.04 | 0.12 | **0.047** |
| rs2162540\|rs10736303\|rs1078806\|rs1219648 | CAAA | 0.04 | 0.13 | **0.034** |
| rs2162540\|rs10736303\|rs1078806\|rs4752566 | CAAT | 0.009 | 0.07 | **0.033** |
| rs2162540\|rs10736303\|rs1078806 | CAA | 0.06 | 0.15 | **0.029** |
| rs2162540\|rs10736303\|rs11200014 | CAG | 0.04 | 0.12 | **0.044** |
| rs2162540\|rs1078806\|rs1219648 | CAA | 0.04 | 0.12 | **0.043** |
| rs2162540\|rs10736303 | CA | 0.08 | 0.18 | **0.043** |
| rs2162540\|rs1078806 | CA | 0.06 | 0.16 | **0.037** |
| rs2162540\|rs11200014 | CG | 0.05 | 0.15 | **0.028** |
| **Mandibular fused roots (94 combinations)** | | | | |
| rs2162540\|rs2981578\|rs10736303\|rs1078806\|rs11200014\|rs1219648\|rs4752566 | TCGAGAT | 0.09 | 0.01 | **0.010** |
| rs2162540\|rs2981578\|rs10736303\|rs1078806\|rs11200014\|rs1219648 | TTGAGA | 0.14 | 0.036 | **0.035** |
| rs2162540\|rs2981578\|rs10736303\|rs1078806\|rs11200014\|rs1219648 | TCGAGA | 0.08 | 0.01 | **0.021** |
| rs2162540\|rs2981578\|rs10736303\|rs1078806\|rs11200014\|rs4752566 | TCGGAT | 0.11 | 0.02 | **0.009** |
| rs2162540\|rs2981578\|rs10736303\|rs1078806\|rs11200014\|rs4752566 | TCGAGT | 0.06 | 0.008 | **0.023** |
| rs2162540\|rs2981578\|rs10736303\|rs1078806\|rs1219648\|rs4752566 | TCGAAT | 0.14 | 0.02 | **0.005** |
| rs2162540\|rs2981578\|rs10736303\|rs1078806\|rs1219648\|rs4752566 | TTGGAG | 0.09 | 0.008 | **0.003** |
| rs2162540\|rs2981578\|rs10736303\|rs11200014\|rs1219648\|rs4752566 | TCGAGT | 0.07 | 0.01 | **0.049** |
| rs2162540\|rs2981578\|rs10736303\|rs11200014\|rs1219648\|rs4752566 | TCGAAT | 0.13 | 0.01 | **0.001** |
| rs2162540\|rs2981578\|rs10736303\|rs11200014\|rs1219648\|rs4752566 | TTGGAG | 0.11 | 0.02 | **0.037** |
| rs2162540\|rs2981578\|rs1078806\|rs11200014\|rs1219648\|rs4752566 | TCGAGT | 0.09 | 0.016 | **0.020** |
| rs2162540\|rs2981578\|rs1078806\|rs11200014\|rs1219648\|rs4752566 | CCGAAT | 0.04 | 0.001 | **0.004** |
| rs2162540\|rs10736303\|rs1078806\|rs11200014\|rs1219648\|rs4752566 | TGGAGT | 0.09 | 0.01 | **0.048** |
| rs2162540\|rs10736303\|rs1078806\|rs11200014\|rs1219648\|rs4752566 | CGGAAT | 0.05 | 0.002 | **0.007** |
| rs2162540\|rs10736303\|rs1078806\|rs11200014\|rs1219648\|rs4752566 | TGAGAG | 0.08 | 0.01 | **0.027** |
| rs2981578\|rs10736303\|rs1078806\|rs11200014\|rs1219648\|rs4752566 | CGAGAT | 0.08 | 0.01 | **0.043** |
| rs2162540\|rs2981578\|rs10736303\|rs1078806\|rs4752566 | TCGAT | 0.10 | 0.01 | **0.009** |
| rs2162540\|rs2981578\|rs10736303\|rs1078806\|rs1219648 | CCGGA | 0.09 | 0.004 | **<0.001** |
| rs2162540\|rs2981578\|rs10736303\|rs1078806\|rs1219648 | TTGAA | 0.13 | 0.03 | **0.038** |
| rs2162540\|rs2981578\|rs10736303\|rs1078806\|rs1219648 | TCGAA | 0.12 | 0.02 | **0.016** |
| rs2162540\|rs2981578\|rs10736303\|rs1078806\|rs11200014 | TCGAG | 0.07 | 0.01 | **0.037** |
| rs2162540\|rs2981578\|rs10736303\|rs1219648\|rs4752566 | TCGAT | 0.20 | 0.03 | **0.001** |
| rs2162540\|rs2981578\|rs10736303\|rs1219648\|rs4752566 | CCGAG | 0.09 | 0.007 | **0.001** |
| rs2162540\|rs2981578\|rs10736303\|rs11200014\|rs1219648 | TCGAA | 0.10 | 0.01 | **0.004** |
| rs2162540\|rs2981578\|rs10736303\|rs11200014\|rs1219648 | TTGGA | 0.16 | 0.04 | **0.017** |
| rs2162540\|rs2981578\|rs10736303\|rs1219648\|rs4752566 | TCGAT | 0.20 | 0.03 | **0.001** |
| rs2162540\|rs2981578\|rs10736303\|rs1219648\|rs4752566 | CCGAG | 0.09 | 0.07 | **0.001** |
| rs2162540\|rs2981578\|rs10736303\|rs11200014\|rs4752566 | TCGAT | 0.16 | 0.03 | **0.005** |
| rs2981578\|rs10736303\|rs1078806\|rs11200014\|rs1219648 | CGAGA | 0.09 | 0.01 | **0.019** |
| rs2981578\|rs10736303\|rs1078806\|rs1219648\|rs4752566 | CGAAT | 0.12 | 0.02 | **0.024** |
| rs2981578\|rs10736303\|rs1078806\|rs11200014\|rs4752566 | CGAGT | 0.06 | 0.01 | **0.044** |
| rs2162540\|rs2981578\|rs10736303\|rs1078806 | TCGA | 0.11 | 0.02 | **0.010** |
| rs2162540\|rs2981578\|rs10736303\|rs1219648 | TTGA | 0.15 | 0.04 | **0.020** |
| rs2162540\|rs2981578\|rs10736303\|rs1219648 | CCGA | 0.09 | 0.01 | **0.008** |
| rs2162540\|rs2981578\|rs10736303\|rs1219648 | TCGA | 0.15 | 0.02 | **0.003** |
| rs2162540\|rs2981578\|rs10736303\|rs11200014 | TCGA | 0.18 | 0.06 | **0.047** |
| rs2162540\|rs2981578\|rs10736303\|rs11200014 | TTGG | 0.14 | 0.04 | **0.045** |
| rs2162540\|rs2981578\|rs10736303\|rs4752566 | TCGT | 0.22 | 0.05 | **0.003** |
| rs2162540\|rs10736303\|rs1078806\|rs11200014 | TGAG | 0.18 | 0.05 | **0.015** |
| rs2162540\|rs10736303\|rs1078806\|rs1219648 | CGGA | 0.09 | 0.003 | **<0.001** |
| rs2162540\|rs10736303\|rs1078806\|rs1219648 | TGGA | 0.07 | 0.01 | **0.025** |
| rs2162540\|rs10736303\|rs1078806\|rs1219648 | TGAA | 0.24 | 0.06 | **0.003** |
| rs2162540\|rs10736303\|rs11200014\|rs4752566 | TGAT | 0.15 | 0.03 | **0.005** |
| rs2162540\|rs1078806\|rs11200014\|rs4752566 | TGAT | 0.09 | 0.01 | **0.005** |
| rs2162540\|rs2981578\|rs11200014\|rs1219648 | TCAA | 0.09 | 0.01 | **0.006** |
| rs2162540\|rs2981578\|rs11200014\|rs4752566 | TCAT | 0.14 | 0.02 | **0.003** |
| rs2981578\|rs10736303\|rs1078806\|rs11200014 | CGAG | 0.07 | 0.01 | **0.033** |
| rs2981578\|rs10736303\|rs1078806\|rs1219648 | CGGA | 0.13 | 0.01 | **<0.001** |
| rs2981578\|rs10736303\|rs1078806\|rs1219648 | CGAA | 0.13 | 0.03 | **0.023** |
| rs2981578\|rs10736303\|rs1078806\|rs4752566 | CGAT | 0.09 | 0.01 | **0.028** |
| rs2981578\|rs10736303\|rs11200014\|rs1219648 | CGAA | 0.13 | 0.01 | **0.001** |
| rs2981578\|rs10736303\|rs11200014\|rs1219648 | TGGA | 0.15 | 0.04 | **0.022** |
| rs2981578\|rs10736303\|rs11200014\|rs1219648 | CGGA | 0.12 | 0.02 | **0.018** |
| rs2981578\|rs10736303\|rs1219648\|rs4752566 | CGAT | 0.14 | 0.03 | **0.012** |
| rs2981578\|rs10736303\|rs1219648\|rs4752566 | TGAG | 0.09 | 0.01 | **0.025** |
| rs2981578\|rs10736303\|rs1219648\|rs4752566 | CGAG | 0.11 | 0.01 | **0.001** |
| rs10736303\|rs1078806\|rs11200014\|rs1219648 | GGGA | 0.09 | 0.01 | **0.022** |
| rs10736303\|rs1078806\|rs11200014\|rs1219648 | GAGA | 0.20 | 0.04 | **0.002** |
| rs10736303\|rs1078806\|rs1219648\|rs4752566 | GAAT | 0.15 | 0.04 | **0.036** |
| rs10736303\|rs1078806\|rs1219648\|rs4752566 | GGAG | 0.13 | 0.01 | **<0.001** |
| rs10736303\|rs1078806\|rs1219648\|rs4752566 | GAAG | 0.09 | 0.02 | **0.046** |
| rs2162540\|rs2981578\|rs10736303 | TCG | 0.25 | 0.09 | **0.021** |
| rs2162540\|rs2981578\|rs11200014 | TCA | 0.18 | 0.06 | **0.044** |
| rs2162540\|rs2981578\|rs4752566 | TCT | 0.17 | 0.05 | **0.024** |
| rs2162540\|rs10736303\|rs1078806 | TGA | 0.23 | 0.06 | **0.006** |
| rs2162540\|rs10736303\|rs1078806 | CAA | 0 | 0.15 | **0.043** |
| rs2162540\|rs10736303\|rs11200014 | TGG | 0.22 | 0.08 | **0.028** |
| rs2162540\|rs10736303\|rs1219648 | CGA | 0.09 | 0.01 | **0.006** |
| rs2162540\|rs10736303\|rs1219648 | TGA | 0.31 | 0.07 | **<0.001** |
| rs2162540\|rs10736303\|rs4752566 | TGT | 0.24 | 0.07 | **0.005** |
| rs2162540\|rs1078806\|rs1219648 | CGA | 0.07 | 0.002 | **<0.001** |
| rs2162540\|rs11200014\|rs1219648 | TAA | 0.09 | 0.01 | **0.032** |
| rs2162540\|rs11200014\|rs4752566 | TAT | 0.13 | 0.02 | **0.005** |
| rs2981578\|rs10736303\|rs1078806 | CGA | 0.10 | 0.02 | **0.033** |
| rs2981578\|rs10736303\|rs11200014 | TGG | 0.14 | 0.04 | **0.039** |
| rs2981578\|rs10736303\|rs1219648 | TGA | 0.15 | 0.04 | **0.021** |
| rs2981578\|rs1078806\|rs1219648 | CGA | 0.12 | 0.01 | **0.004** |
| rs2981578\|rs11200014\|rs1219648 | CAA | 0.13 | 0.01 | **0.001** |
| rs1078806\|rs11200014\|rs1219648 | GAA | 0.09 | 0.005 | **<0.001** |
| rs1078806\|rs1219648\|rs4752566 | GAG | 0.13 | 0.02 | **0.013** |
| rs2162540\|rs10736303 | TG | 0.40 | 0.15 | **0.002** |
| rs2162540\|rs10736303 | CA | 0 | 0.18 | **0.028** |
| rs2162540\|rs10736303 | TG | 0.14 | 0.04 | **0.048** |
| rs2981578\|rs10736303 | TG | 0.31 | 0.14 | **0.048** |
| rs2981578\|rs1219648 | CA | 0.31 | 0.14 | **0.037** |
| rs10736303\|rs1078806 | GA | 0.22 | 0.06 | **0.007** |
| rs10736303\|rs11200014 | GG | 0.27 | 0.09 | **0.011** |
| rs1078806\|rs1219648 | GA | 0.18 | 0.05 | **0.017** |
| rs11200014\|rs1219648 | AA | 0.13 | 0.03 | **0.017** |
| rs2981578\|rs10736303 | TG | 0.14 | 0.04 | **0.048** |
| rs2981578\|rs1219648 | CA | 0.31 | 0.14 | **0.037** |
| rs10736303\|rs11200014 | GG | 0.27 | 0.09 | **0.011** |
| rs1078806\|rs1219648 | GA | 0.18 | 0.05 | **0.017** |
| rs11200014\|rs1219648 | AA | 0.13 | 0.03 | **0.017** |
