# Supplementary material for: Fibroblast growth factor receptor 2 (FGFR2) genetic polymorphisms contribute to fused roots in human molars
Source: PLoS One. 2025 Apr 10;20(4):e0316904. doi: 10.1371/journal.pone.0316904 (PMC11984737; doi:10.1371/journal.pone.0316904)
Supplement: Attachment — Submitted filename: Response to Reviewers.docx [file pone.0316904.s003.docx]

**Response to Reviewers**

Dear Sonam Khurana

Academic Editor, Plos One

Manuscript ID:

PONE-D-24-26278 entitled "Fibroblast growth factor receptor 2 (FGFR2) genetic polymorphisms contribute to fused roots in human molars" previously submitted to Plos One was revised and rewritten. This new manuscript has considered the reviewers' comments. The authors followed all their suggestions and included the answers to their statements in this letter.

We hope we have achieved your expectations.

With kind regards,

The authors

Reviewer's Responses to Questions:

Journal Requirements:

1. When submitting your revision, we need you to address these additional requirements.

Please ensure that your manuscript meets PLOS ONE's style requirements, including those for file naming. The PLOS ONE style templates can be found at

https://journals.plos.org/plosone/s/file?id=wjVg/PLOSOne_formatting_sample_main_body.pdf and

<https://journals.plos.org/plosone/s/file?id=ba62/PLOSOne_formatting_sample_title_authors_affiliations.pdf>

R;. Thank you. Requirements checked.

2. Thank you for stating the following in the Acknowledgments Section of your manuscript:

We are indebted to all participants. This work was supported by the Open Access Publication Fund of the University of Bonn. This study was financed in part by the Alexander-von-Humboldt-Foundation. This study was financed in part by the Coordenação de Aperfeiçoamento de Pessoal de Nível Superior - Brasil (CAPES) - Finance Code 001.

We note that you have provided funding information that is not currently declared in your Funding Statement. However, funding information should not appear in the Acknowledgments section or other areas of your manuscript. We will only publish funding information present in the Funding Statement section of the online submission form.

Please remove any funding-related text from the manuscript and let us know how you would like to update your Funding Statement. Currently, your Funding Statement reads as follows:

The author(s) received no specific funding for this work.

*R:. Thank you. We have removed the funding from the Acknowledgments section and inserted in the Funding section*

*Funding*

*This work was supported by the Open Access Publication Fund of the University of Bonn. This study was financed in part by the Alexander-von-Humboldt-Foundation. This study was financed in part by the Coordenação de Aperfeiçoamento de Pessoal de Nível Superior - Brasil (CAPES) - Finance Code 001.*

Please include your amended statements within your cover letter; we will change the online submission form on your behalf.

*R:. We included. Thank you.*

3. Thank you for stating the following in your Competing Interests section:

"None"

Please complete your Competing Interests on the online submission form to state any Competing Interests. If you have no competing interests, please state "The authors have declared that no competing interests exist.", as detailed online in our guide for authors at http://journals.plos.org/plosone/s/submit-now

This information should be included in your cover letter; we will change the online submission form on your behalf.

*R;. . Thank you.*

Reviewers' comments:

Reviewer #1: The study aims to explore whether genetic polymorphisms in FGFR2 are associated with fused roots in human molars, which is a valid research objective given the gene's known role in craniofacial development. However, there's some points to clarify:

1- Ensure that the study has obtained appropriate ethical approvals and informed consent from participants. This is crucial for studies involving human subjects. as you didn't submit any ethics statement even it's included in the Methods section of the manuscript.

*R:. Thank you. We attached the ethics statement in the submission.*

2- The article reports associations between FGFR2 SNPs and fused roots, but does not delve deeply into the biological mechanisms. Adding a discussion on how these genetic variations could affect dental development would strengthen the argument.

*R:. Thank you. We performed some alterations in the discussion section as suggested by all the reviewers.*

3 - Ensure the sample size calculation aligns with the study’s goals and statistical power. Confirm that the chosen sample size is adequate for detecting significant associations this will help to discuss the extent to which the findings can be generalized to other populations. If the sample is not representative of the broader population, this should be acknowledged.

*R:. Thank you. A sample size calculation was performed for this study with a power of 80% and alpha of 5% using the data found in Ross & Evanchik (1981) in which observed a frequency of 29% of fused root in all molars. Therefore, the sample size was calculated in the case to control ratio of 1:3 assuming a 25% difference between the genotypes. The estimation for the total sample of 156 patients were required (case to control ratio 39:117). In the present study a total of 170 patients were included.*

Reference: Ross IF, Evanchik PA. Root fusion in molars: incidence and sex linkage. J Periodontol. 1981;52(11):663–7.

Reviewer #2: The authors conducted a good study about FGFR2 and his role efforts on the fused roots of molars. The study used good sample size and Investigative of FGFR2 in well method. Really, I enjoyed reading this article.

*R:. Thank you very much for the positive assessment of our work.*

Reviewer #3: There is interesting information presented in this paper and does contribute to the advancement of the field. Overall, though, this paper is missing a developed discussion about why the authors chose to assess FGFR2, rather than a different gene, in conjunction with fused roots in humans. The absence of this information is notable in the introduction, discussion and conclusion sections.

*R:. Thank you for your comment. We added several new information in the introduction, a paragraph justifying our choice of FGFR2 gene (highlighted in yellow).*

The presented hypothesis is also not clear and justification is missing.

*R:. Thank you. We have rewritten and added more information to a better comprehension of the hypothesis. Syndromic cases of dental root fusion are observed in patients with FGFR2 mutations. FGFs signaling are required for human tooth development and its dysregulation seriously affects tooth development in humans, leading to tooth anomalies. Therefore, it is reasonable to hypothesize that genetic variations in FGFR2 are also account for isolated cases (non-syndromic) of dental fused roots, which is a dental development alteration common in human molars. Thus, the aim of this study was to investigate whether genetic polymorphisms in FGFR2 are associated with molar fused roots phenotype.*

There is missing information in descriptions of sample selection and methods, and presentation of some statistical analyses. Sections of this paper could also be reorganized to provide better context and readability.

*R:. Thank you. We reorganized the methods and also the results*

Abstract: The abstract is missing a reference to the hypothesis presented in the paper or specifics of the sample analyzed.

*R:. Thank you. We inserted the hypothesis and detailed the sample in the abstract.*

Line 50-52: What specific comparisons are the p-values referring to?

*R:. Thank you. The significance level was set at p < 0.05 for all the analysis. We have performed several corrections in the abstract.*

Line 54: The word "risk" is used in reference to OR. Since OR is a probability, this test is not assessing the risk, but rather than chance of having fused roots. Consider changing the wording here and elsewhere in the paper (see comments below).

*R:. Thank you for your comments. We have made the changes in the whole manuscript.*

Introduction: The introduction does not outline the point of the study very clearly or provide a clear hypothesis being tested.

*R;. Thank you. We have rewritten the hypothesis in the introduction.*

Information in paragraph 2 (starting on line 67) could be better served as the leading paragraph to contextualize the study. Lines 62-66 could then be integrating into paragraph two of the introduction. While literature is mentioned in this introduction, no substantive background information or specific examples are provided.

*R;. Thank you. We have rewritten the introduction.*

Line 64-66: There are several stages of tooth development, how does this relate to root development specifically?

*R;. Thank you. The introduction was re-written after all reviewers’ comments.*

Paragraphs 7-9 from the discussion section (there are no line numbers in the discussion section, so paragraph numbers are used as a reference) provide some of the background information that would contextualize this study. I suggest integrating those paragraphs into the introduction and deleting them from the discussion section.

*R.: Thank you. We have integrated those paragraphs in the introduction as suggested. We have also inserted lines in the discussion.*

Line 79-82: Is this the hypothesis you are testing? There is no justification for why dental anomalies would be associated with non-syndromic cases. Please provide the background for this. In the subsequent line, this study sounds like an exploratory study rather than hypothesis testing. Please outline specific expectations with background justification if this is a hypothesis-testing study.

*R.: Thank you. The hypothesis is the polymorphisms in FGFR2 are involved in fused roots also in non-syndromic patients, as observed in other studies (for example with oral cleft or tooth agenesis) in which the syndromic form brings some clue to investigate the isolate form of an anomaly. We rewrote this part in order to clarify for the readers.*

Materials and Methods: Sample breakdowns are not presented in the materials section. This is information is provided in the results section (168-172). Integrate that information within lines 92-101 and delete in the results section. Specific information about participant selection and identification of fused roots needs clarification.

*R;. Thank you for your comments and suggestion. We have added more information of the samples and integrated the information of the results in methods section as suggested.*

Line 96: Define how you determined "case" and "control" and how many of each you have in this study.

*R;. Thank you. We have added a sentence at the end of Methods:* ***Evaluation of fused roots phenotype:*** *upper and lower first and second molars were assessed for presence or absence of root fusion, and the patients included in case (molars with fused roots) and control (molars without fused roots) groups*.

Lines 102-108: You mention not using third molars. Specify which teeth you are analyzing for this study.

*R:. Thank you. We included in this study* *upper and lower first and second molars.*

*“Based on these radiographic parameters, the upper and lower first and second molars were assessed for presence or absence of root fusion.”*

Line 103-106: Was there consideration to eruption stage or an age cut-off set to select participants? Please be specific about the criteria for participation.

*R.: Thank you. As stated in the inclusion and exclusion criteria the patients should present the first and second upper and lower permanent molars, without immature roots. Third molars were not considered, since the root of these teeth is not completely formed at the initial age of recruitment. Additionally, this is a region more difficult to visualize.*

Line 109-110: Which author(s) performed analyses? Please also indicate whether or not data collectors were blinded to group membership (the "case" and "control" groups). What protocol was used for identifying fused molar roots? Is this the same as information from lines 117-120?

*R.: Thank you. Locks MEN, performed the panoramic analyses and was blinded to the groups/patients and did not participate in the genotyping analysis. The protocol was based in the study of Ross & Evanchik. We have stated this in the methodology*.

Line 117-118: How was "typical furcal position" defined? Since maxillary and mandibular roots are different, how did this definition change between the two? Please be specific about how you determined this.

*R:. Thank you. The criteria were based on a previous published paper (Ross & Evanchik) in which*

*any molar that had one root or whose roots were fused apical to the usual furcal position was considered a molar with fused roots. This included molars with fusion of one-third or less of the roots and molars with fusion of entire root surfaces. A tooth was considered fused regardless of location of the fusion. Fusion could occur in the apical one-third, middle one-third, or cervical one-third of the roots or in any combination of two or more of these areas. In some cases, molars had roots fused only in the apical one-third and had a normal furcation with alveolar bone and other periodontal structures present. These teeth were included in the category of fused teeth to avoid misunderstanding and inconsistency.*

*We have included this part in the* *“Evaluation of fused roots phenotype” section.*

*Reference: Ross, I. F., & Evanchik, P. A. (1981). Root Fusion in Molars: Incidence and Sex Linkage. Journal of Periodontology, 52(11), 663–667. doi:10.1902/jop.1981.52.11.663*

Line 124: The inclusion of a literature search is not clear. It seems that this is how the genetic analysis was selected, but the use of a literature search is not well developed here.

*R:. Thank you. We re-wrote this topic to clarify that the literature search was performed in order to allow the identification of the candidate gene.*

Table 1: Please contextualize the Note at the bottom of the table. Where did you integrate this information? How does this relate to the literature search?

*R:. Thank you. We made the contextualization as requested and we inserted the references to integrate the information.*

Line 145: Provide the specific statistical comparisons that were run for chi-squared, OR, and CI.

*R:. Thank you. We added the information in the methods “Odds ratios (OR) and 95 % confidence intervals (CI) were calculated to evaluate the chance of presenting fused roots in the allelic discrimination analysis.” We also organized the results to clarify the different analysis.*

Results: Some statistical analyses seem to be missing; OR and CI were only run for a handful of comparisons and those comparisons are not clear. Please include OR and CI in a table with the comparisons they correspond to. It is unclear which comparisons the p-values in the text refer to. Adding a breakdown of the specific comparisons in the statistical analysis section will help with this, but it should also be clear in the results section. Just a note, S2 Table is referenced before S1 Table in the text. Please change.

*R: Thank you for your relevant consideration. We have made some changes. We included a separated table (table 4 with the detailed OR and CI, that was before the S2) and have rewritten the results for a better comprehension, once they were calculated only for allelic discrimination analysis.*

Line 156: This section would be better served in the Materials and Methods section when discussing the literature review. It seems to be more background information rather than results from an analysis. Because this paper is not presented as a meta analysis, this section does not fit in the results section.

*R:. Thank you. We agree with your comments. We removed this section from the results and placed in the new topic of the method.*

Fig 2 caption: The wording of this caption is not clear. Suggested change to something similar to: schematic summarizing possible morphological variations of fused roots found from literature on patients presenting mutation in FGFR2.

*R:. Thank you. We have changed the wording of Fig 2 caption as suggested.*

Line 168: Suggest moving this section on sample description to materials section since it is not presenting results.

*R.: Thank you. We have rewritten this section.*

Table 2: Please specify what "reference" refers to.

*R.: We added this information in both tables to explain the reference is the control group that was used for the comparisons of all analysis.*

Line 182: Please provide OR for the other comparisons presented so the reader can see the differences.

*R.: Thank you. OR for all comparisons are presented now in Table 4.*

Line 187-189: Change "risk" to "probability" or "chance". Please provide OR for all comparisons presented so the reader can see the differences.

*R.: Thank you. We have changed the word “risk” to “chance” in the whole manuscript.* ". *OR for all allelic distribution comparisons are presented now in Table 4.*

Line 189: S2 Table is referenced as showing higher probability of having fused roots with at least one C allele, but probability results (OR) are not presented in S2 Table.

*R.: Thank you. We have adjusted the data.*

Discussion: Overall, the flow in this section is hard to follow. More specific examples are needed to contextualize how this study fits with previous literature. It remains unclear why exactly FGFR2 was picked for this study. This section does address how the current study may add to advancing the field but this discussion is not well developed. The term "dental anomalies" is a large category; in this section there should be more specific reference to root fusion since that is the focus of the paper. The last several paragraphs of this section do not discuss FGFR2 or the results of this study and would be better served in the introduction and methods sections. Note: line numbers are absent in this section; paragraph numbers are used as a reference:

Paragraph 1, Sentence 2: This contradicts the reference you make to a hypothesis in the introduction. Please change for consistency. As stated above, there needs to be more background information to justify these statements.

*R.: Thank you. We have made the changes in the introduction and 1^st^ paragraph of discussion for consistency*

Paragraph 2: This paragraph does not flow with the previous or subsequent. Suggest integrating this information into paragraph 4.

*R.; Thank you. We have made the integration of these paragraphs as requested.*

Paragraph 4: This feels like a continuation of paragraph 3. Consider combining them.

*R.; Thank you. We have made the integration of these paragraphs as requested.*

Paragraph 4, Sentence 5: Provide some specific examples of root developmental anomalies to distinguish from root fusion.

*R.: Thank you. We have given some examples of dental development anomalies*

Discussion, Paragraph 5, Sentence 4: You say "interactions that oversee the progression of morphogenesis and histodifferentiation of the tooth". How would this relate to root development specifically?

*R.: We rewrote this part.*

Paragraph 5, Sentence 5-9 and 11: Provide specific examples. Did any include root anomalies (specifically fusion)?

R.: *Thank you. We have given some examples of dental development anomalies*

Paragraphs 7: This information should be included as background in the introduction that leads to the hypothesis. I suggest moving it there and deleting from the discussion section.

*R.; Thank you. We have included this information in the introduction*

Paragraph 8: While true, this information does not provide any context for the present study. Consider removing or integrating within the context of the current study.

*R.: Thank you for your consideration. We have integrated this part in the introduction*

Paragraph 9: This information should be included as background in the introduction that leads to the hypothesis. I suggest moving it there and deleting from the discussion section.

*R.; Thank you. We have made the modification.*

Paragraph 10: This information should be included in the materials and methods section or introduction. I suggest moving it and deleting from the discussion section.

*R.; Thank you for your comment. According to the STREGA, the limitations of the study should be placed in the discussion section.*

Conclusion: This section is just a reiteration of the last sentence in the abstract. Please add a brief summary and more context about the significance if you keep this section.

*R.: Thank you. We have rewritten the conclusion.*

Reviewer #4: This manuscript needs minor revision. See my comments below to improve this manuscript.

- introduction

Please explain more about the FGFR2 mutations and functions in dental studies based on literature review.

*R.; Thank you. We have included this information in the introduction*

- Discussion:

1. second paragraph: “FGFR2 is a highly …. of several signal transduction pathways such as …. that are crucial for osteogenic differentiation [22].” please mention these pathways.

*R:. Thank you. We have included the main pathways as requested (RAS/mitogen-activating protein (MAP) kinase, the phosphoinositide 3 (PI3) kinase/ AKT, and the phospholipase C gamma (PLC Ƴ)*

2. Please write “Fgf” in capital and mention a list of FGF functions except tooth morphogenesis.

*R:. Thank you. We have included other functions of FGF as requested, such as cellular proliferation, differentiation, and migration, and patterning.*

3. Please explain how the interaction affects FGFR2. You explained the effect of each one separately on tooth development “The interaction between RUNX2 and SMAD4 was observed to enhance the expression of FGFR2”.

*R:. Thank you. We added more information in this sentence.*

- Please extend the conclusion part.

*R.: Thank you. We have extended the conclusion*

Reviewer #5: The manuscript submitted titled 'Fibroblast growth factor receptor 2 (FGFR2) genetic polymorphisms contribute to fused roots in human molars' presents interesting results and has substantial merit. There are some minor suggestions.

In abstract, the abbreviation SNP is not introduced.

*R:. Thank you. The abbreviation was firstly introduced in the abstract as requested.*

On page 13, the abbreviations of fgf and smad4 are not as described elsewhere so to make the style consistent, it is suggested to use FGF and SMAD4.

*R:. Thank you. We have made the changes for FGF accordingly. In the case of Smad4, in animal studies, the genes are written in lower case.*

In methodology, how the cases and controls were selected to be a part of the study is not clearly mentioned. It is suggested to include the sampling technique used. Any potential bias also needs to be stated.

*R.: Thank you. They were not selected. All patients that met the inclusion/exclusion criteria were included. We added the information that this was a nested cross-sectional (“nested cross-sectional phenotype-genotype association study”) and the recruitment process (“we recruited a convenience sample of consecutively orthodontic patients”) in the methods.*

Some references in the reference list needs revision where titles are not in sentence case.

*R:. Thank you. We have revised the reference list.*

Reviewer #6: Meyfarth et el., investigated the association between genetics and fused roots in human molars. Based on existing literature, they selected FGFR2 for analysis. The authors analyzed panoramic radiographs from 170 orthodontic patients to identify fused molar roots, and genotyped 7 single nucleotide polymorphisms (SNPs) in FGFR2. They found significant associations between two SNPs (rs10736303 and rs2162540) and the presence of fused molar roots. Individuals carrying certain alleles of these SNPs had increased odds of presenting with fused roots. The authors conclude that genetic variation in FGFR2 may contribute to the development of fused roots in human molars. This study is well designed and well performed, adding more support to the role of FGFR2 in tooth development, and as such is suitable for publication in PLoS one, after addressing a few issues.

Major comments:

1. Genotyping SNPs using real-time PCR can be challenging. The authors should include data showing their ability to successfully genotype using this method, and provide a detailed protocol including primer sequences.

*R:. Thank you. We have detailed the genotyping protocol as requested and explained how we checked the consistence. Primer sequence is not available, once we ordered pre-designed probes/primers from Applied Biosystems, but we added at table 1 the ID of each SNP.*

2. Multiple testing: The authors tested multiple SNPs and models without apparent correction for multiple comparisons. This increases the risk of type I errors. The authors should apply and discuss appropriate corrections.

*R:. Thank you for your valuable comment. We discussed the error type I limitation in this new version. We did not apply any correction to avoid error type II, once the sample size calculation was performed with an alpha of 5%. See comments at the end of the discussion section regarding limitations.*

3. The authors should provide a detailed explanation and/or reference for the dominance models used.

*R:. Thank you. We added a sentence in the statistical analysis of the method to explain the models.*

Minor comments:

4. The introduction could be expanded to provide more background on the biology of root development and the role of FGFR2.

*R;. Thank you. We added more information in the introduction as requested.*

5. The results section would benefit from a table summarizing the demographic characteristics of the sample.

*R.: Thank you. We have included a table as requested (Table 2)*

6. The discussion could be strengthened by more thoroughly exploring the potential functional consequences of the identified SNPs and how they may influence root development.

*R:. Thank you. Several alterations were performed in the discussion.*

7. Blinding in phenotype assessment: The authors do not specify whether the examiner evaluating the panoramic radiographs for fused roots was blinded to the genetic data or other participant information.

*R.: Thank you.* *Locks MEN, performed the panoramic analyses and was blinded to the groups/patients and did not participate in the genotyping analysis. We have stated this in the methodology*.
