# Supplementary material for: Fibroblast growth factor receptor 2 (FGFR2) genetic polymorphisms contribute to fused roots in human molars
Source: PLoS One. 2025 Apr 10;20(4):e0316904. doi: 10.1371/journal.pone.0316904 (PMC11984737; doi:10.1371/journal.pone.0316904)
Supplement: Attachment — Submitted filename: Response_to_Reviewers_auresp_2.docx [file pone.0316904.s004.docx]

**Response to Reviewers**

Dear Gary S. Stein

Academic Editor, Plos One

Manuscript ID:

PONE-D-24-26278R1 entitled "Fibroblast growth factor receptor 2 (FGFR2) genetic polymorphisms contribute to fused roots in human molars" previously submitted to Plos One was revised and rewritten. This new manuscript has considered the reviewers' comments. The authors followed all their suggestions and included the answers to their statements in this letter.

We hope we have achieved your expectations.

With kind regards,

The authors

Reviewers' comments:

Review Comments to the Author

Please use the space provided to explain your answers to the questions above. You may also include additional comments for the author, including concerns about dual publication, research ethics, or publication ethics. (Please upload your review as an attachment if it exceeds 20,000 characters)

Reviewer #1: The authors have carefully taken into consideration all the comments and suggestions I provided them with. As a result, the article is now much more suitable and appropriate for potential publication in a scientific journal (Q1).

*R.: Thank you very much.*

Reviewer #2: I'm stratified with the current version of the manuscript. The authors declare all the required informations. I recommend it for publication.

*R.: Thank you very much.*

Reviewer #3: The authors have done a great job of addressing the feedback they were given. This updated version has a clear purpose with justified arguments and relevant supporting examples. I appreciate the work the authors put in to revise this manuscript. My comments are minor.

*R.: Thank you very much.*

Overall, the abstract reads well. As it currently reads, lines 40-42 place emphasis on the literature review search instead of the data collection done for the study. Suggest reworking these lines or deleting them. The rest of the abstract is clear.

*R.: Thank you. We have rewritten these lines as suggested.*

Line 285: Hypotheses are "supported" or are "failed to reject" please change wording from "confirmed"

*R.: Thank you. We removed the sentence.*

Line 341-344: While I appreciate the decision not to use post-analysis adjustments, this section reads awkwardly. Sources such as Perneger 1998; Cabin and Mitchell 2000; Nakagawa 2004; Garcia-Perex 2023 could be cited to strengthen the argument for avoiding type II errors by not using adjustments. Consider reworking this section for clarity and supporting arguments.

*R.: Thank you for your consideration. We rewrote the sentence and added the Perneger as a reference for our choice to do not perform Bonferroni correction.*

Reviewer #4: This manuscript is interesting and needs some modifications.

Page 2 line 39: Please delete the second “that”.

*R.: Thank you. It was deleted.*

Page 2 line 55 and 162: Please delete”, “before type 2.

*R.: Thank you. It was deleted.*

Page 16 line 308: Whatever you explained in line 308 is dependent on SMAD. Based on literature, please explain how FGFR2 and RUNX2 are related together without the SMAD4 pathway and add it to the text.

*R.: Thank you. We have added this information as requested.*

Page 17 line 316: Is SMAD4 activation through TGF-b in your study? Please add the reference for that (based on literature).

Thank you very much.

Best regards

R.: *Thank you. In our study we did not make this evaluation, but we added in the discussion a suggestion for future studies.*

Reviewer #5: Authors have incorporated the changes that were suggested. However, there is a minor correction required in Table 1. In Assay ID* and Base Change*, asterisk is usually for significant p values, it is suggested to use some other symbol to avoid confusion.

*R.: Thank you. We have changed as suggested.*

Reviewer #6: The authors addressed much of the concerns, but unfortunately did not fully address the two most important ones - assay QC and statistical validity.

1. While providing catalog numbers instead of sequences can be reasonable, quality controls still must be included, optionally as supplementary material. Merely repeating a subset of experiments, validating the consistency but not the correctness, is insufficient. Controls can include VIC/FAM intensities over time for known control DNA samples, as well as the cutoff thresholds. This is particularly important as the reaction volume (3ul) is significantly below the recommended range for the reaction mix used.

*R.: Thank you for your thoughtful and detailed feedback. We acknowledge the importance of additional quality control metrics. Incorporating VIC/FAM intensities over time for known control DNA samples and detailing cutoff thresholds are excellent suggestions that we will consider for future supplementary materials. Regarding the reaction volume, we recognize that 3 µL is below the standard recommendation for the reaction mix. However, this volume was carefully optimized for our experimental conditions to ensure accuracy and consistency, supported by internal validations. This was added in the method section.*

2. Not applying multiple-testing correction as it may eliminate some true results, while potentially introducing many false ones, is not a reasonable strategy to overcome sample-size power limitations. P-values are meaningless without such a correction. The authors may show unadjusted p-value alongside the adjusted one, for “suggestive” hits, but without passing that threshold, there is no statistical confidence in these results, and the manuscript should reflect that.

*R,: Thank you. We opted for not perform a multiple-testing correction. This was explained in the discussion section in more details.*

minor:

“All DNA samples presented a 260/280 ratio lower than 1.8.” - That is neither informative nor reassuring. The ratio should be ~1.6 to 2.0.

*R,: Thank you. We re-wrote this sentence.*

The DNA evaluation section repeats twice, deviating on the number of ul used to test.

*R.: Thank you for your consideration. We have excluded the duplicated sentences and made the correction regarding the µL used that was 2 µL.*
